# Editorial: Veterinary Reproductive Immunology

**DOI:** 10.3389/fvets.2021.823169

**Published:** 2022-01-10

**Authors:** Dariusz J. Skarzynski, Fuller W. Bazer, Juan G. Maldonado-Estrada

**Affiliations:** ^1^Department of Reproductive Immunology and Pathology, Institute of Animal Reproduction and Food Research, Polish Academy of Science, Olsztyn, Poland; ^2^Department of Animal Science, Texas A&M University, College Station, TX, United States; ^3^OHVRI Research Group, Escuela de Medicina Veterinaria, Universidad de Antioquia, Medellín, Colombia

**Keywords:** domestic animals, endometrium, fetal allograft, innate immune cells, Medawar paradox, pregnancy loss, reproductive immunology, T-regulatory cells

## From Medawar's Paradox to Neuroimmunoendocrine, Metabolic and Environmental Integration

The field of Reproductive Immunology has evolved from the paradigms of transplantation immunology to an integrated concept of interactions between the endocrine, immune, nervous, and reproductive systems as critical components of the physiology of reproduction. The origin of reproductive immunology was the Medawar paradox when he proposed several theories explaining failure of the mother to reject the fetal allograft in an epoch when the rules of Major Histocompatibility Complex and its compromise in transplantation immunology were defined. From an orthodox immunologist's perspective, it is hard to fully understand the mechanisms underlying maternal-fetal tolerance in mammalian reproduction. Medawar's theories were tested experimentally by several scientists, whose results are considered fundamental to the origin of reproductive immunology. Although Medawar' paradox is considered the inflection point for the origin of reproductive immunology (1, 2), Billington (3) stated that Mechnikov and Landsteiner were the pioneers of reproductive immunology with their discoveries of expression of phagocytic cells and Fc receptors in placentae that are responsible for uptake of placental antibodies in women during gestation. Also, there was the discovery of hemolytic anemia in the rhesus monkey and its treatment with counteracting antibodies, respectively (3). Since the first hypotheses proposed by Medawar on the possible mechanisms of non-rejection of the fetal allografts, to the pioneering studies in rodents and in women undertaken by Beer, Billingham, Scott, and Yang on maternal-fetal tolerance in the 60's (3), pioneers in reproductive immunology have paved the way for current research in reproductive immunology of human and other mammals.

Regarding the compromise of MHC antigens in reproduction, Billington reported that fetal size was greater when female mice produced fetuses from a strain different from their own (4). In this paper, Billington cited early work highlighting the importance of fetal antigens and cells in establishing maternal-fetal tolerance in mice, rats, cattle, and humans. In that epoch, the first report on circulating syncytiotrophoblast fragments in human pregnancy and their possible relationship to maternal tolerance to the fetal allograft (5) provided further evidence of for a relationship to pathologies such as preeclampsia (6).

Several pieces of evidence support the role of fetal MHC antigens in successful pregnancies, depending on the species and stage of gestation (7–12). The presence of maternal cells in the fetal circulation and fetal cells and cell-free DNA trafficking into the maternal circulation was reported for humans [(13–15), and reviewed in (16)], mice (17–19), and domestic animal species (20–22). The trafficking of cells between the mother and the fetus is a crucial component of the interaction between maternal and fetal immune cells (13, 23–25), highlighting the importance of maternal cells in “educating” the fetal immune system for the antigenic environment it will face in postnatal life [(26), reviewed in (27, 28)]. The presence of maternal immune cells at the maternal-fetal interface is accepted as a critical component of the physiology of gestation (29) and in pathological conditions such as hypertensive disorders of pregnancy, including preeclampsia (30–32).

Several authors proposed that fetal cells bearing MHC antigens and other circulating fetal antigens could act as conventional triggers of the maternal cellular immune responses resulting in rejection of the fetal allograft and maternally induced runt disease in the offspring (33, 34). Accordingly, the work of led by Clark and Chaouat with the CBA2/DBA model in the 1980s (35–37) provided evidence of immune-mediated fetal resorption. This model helped test several hypotheses on the compromise of stressful conditions in the physiopathology of immune-mediated pregnancy loss.

In the late 1970s and early 1980s, research by Martal et al. (38, 39) and Bazer and Roberts group on ovine and bovine trophoblast interferons established the molecular basis of maternal recognition of pregnancy in ruminants (40–42) and triggered an exciting field of research in the maternal-fetal dialogue between the endometrium, the corpus luteum, and the hypothalamic-hypophyseal axis.

The work led by Anne Croy showed the essential role that the trophoblast layer of the placenta plays in maintaining interspecies pregnancies (43). Furthermore, Croy's team (44) and Moffett's group (45, 46) provided evidence for the importance of uterine Natural Killer cells, dendritic cells (47), and innate uterine lymphoid cells [reviewed in (48)] for successful pregnancies in mice and humans, highlighting the importance of innate immune cells in the physiology of gestation. Besides, the works by Antczak and Allen on the compromise of the maternal immune response in the developing chorionic girdles in equine placentation in the early 80's (49, 50) provided evidence for the importance of cells of the adaptive immune system in successful placental development and fetal growth in mares.

The proposal of an immunotropic hypothesis (51) elicited new and exciting concepts to the field of reproductive immunology [(52, 53), reviewed in (54, 55)] and elicited controversy (56–58) on the importance of the adaptive immune system in gestation maintenance. The initial concept of a Th1/Th2 balance required for successful gestation further evolved toward the concept of a balance between regulatory T cells (T-reg) (59) and T-reg/Th17 cells being critical for successful pregnancies (60, 61).

From the beginning of the twenty-first century, several studies by Skarzynski's group provided evidence for a neuro-immuno-endocrine interaction between the endometrium and corpus luteum in cattle (62–67) and horses (68, 69) and the role epithelial cells play in the maternal-fetal dialogue (70). Concomitantly, there were results from Bazer's group on the compromise of several modulators in the maternal-fetal dialogue in sheep (71–75) add critical evidence regarding the function of the reproductive system as an integrated system in which neuro-immune-endocrine and metabolic cues are integrated for successful reproduction (or failure if loss of homeostasis). Other research groups around the world provided additional evidence for the importance of cellular and humoral immune components and processes in the physiology of pregnancy (54, 76–79). Failure in several processes were implicated in immune-mediated embryonic and fetal losses during the course of gestation [reviewed in (80)].

In this special edition on Veterinary Reproductive Immunology, several papers contributed to increasing our understanding of neuro-immune-endocrine interactions related to physiological and pathological conditions of gestation. The papers provide novel results related to anti-GnRH vaccines in cats, the establishment of pregnancy in dogs, processes related to endometrial function in cattle, horses, and pigs, and pre-implantation signaling in mice. Further, the papers included address state-of-the-art protocols in molecular biology, providing readers, scientists, and clinicians with advanced concepts on reproductive immunology. The discussion is still open, as mentioned by Billington (3), who proposed that reproductive immunology would continuously provide scientific information and controversy (3), as it is an essential aspect of research and discovery in the field of animal reproduction. New research areas, including glycan expression at the maternal-fetal interface in the placenta of several animal species and humans, are becoming more prominent in reproductive immunology. However, there are still a lack of comprehensive theories integrating the contributions of findings from studies in glycobiology into concepts related to successful mammalian reproduction. Even though there is abundant scientific evidence for the essential roles that signals from immune, endocrine, nervous, epithelial, stromal, and trophoblast cells produce to intercommunicate the endocrine, immune, neural, and reproductive tissues, no explicit theories exist to fully explain the way these systems function and contribute to maintaining the physiology of gestation or why, when the system is altered, there are losses of gestations. For these reasons, in the future, we will seek momentum in research whereby the scientific community will provide an integrated view on the homeostasis required for successful reproduction, including integration of immunology, genomics, proteomics, glycomics, and environmental influences in mammalian reproduction.

## Author Contributions

DS discussed and corrected the final version of the manuscript. All authors contributed to the article and approved the submitted version.

## Funding

This work was supported by Etrategia de Sostebibilidad de Grupos, CODI, Universidad de Antioquia to JM-E and internal grant of IARFR PAS No 5/FBW/2021 supports to DS. FB was also supported by Grant Arginine and secreted phosphoprotein 1 mediate cell signaling to enhance conceptus development and survival from the National Institute of Food and Agriculture (NIFA) as Competitive Grant no. 2016-67015-24958 from the USDA National Institute of Food and Agriculture and Roles of fructose and glucose in growth and development of ovine and porcine conceptuses, Competitive Grant No. 2018-67015-28093.

## Conflict of Interest

The authors declare that the research was conducted in the absence of any commercial or financial relationships that could be construed as a potential conflict of interest.

## Publisher's Note

All claims expressed in this article are solely those of the authors and do not necessarily represent those of their affiliated organizations, or those of the publisher, the editors and the reviewers. Any product that may be evaluated in this article, or claim that may be made by its manufacturer, is not guaranteed or endorsed by the publisher.
